# Fluvastatin attenuates hepatic steatosis-induced fibrogenesis in rats through inhibiting paracrine effect of hepatocyte on hepatic stellate cells

**DOI:** 10.1186/s12876-015-0248-8

**Published:** 2015-02-15

**Authors:** Lee-Won Chong, Yi-Chao Hsu, Ting-Fang Lee, Yun Lin, Yung-Tsung Chiu, Kuo-Ching Yang, Jaw-Ching Wu, Yi-Tsau Huang

**Affiliations:** 1 Institute of Clinical Medicine, School of Medicine, National Yang Ming University, Taipei, Taiwan; 2 Division of Hepatology and Gastroenterology, Department of Internal Medicine, Shin Kong Wu Ho-Su Memorial Hospital, Taipei, Taiwan; 3 Institute of Biomedical Sciences, Mackay Medical College, New Taipei City, Taiwan; 4 National Research Institute of Chinese Medicine, No. 155-1, Li-Nong Street, Sec. 2, Taipei, 112 Taiwan; 5 Department of Medical Research and Education, Taichung Veterans General Hospital, Taichung, Taiwan; 6 Translational Research Division, Medical Research Department, Taipei Veterans General Hospital, Taipei, Taiwan; 7 Institute of Traditional Medicine, School of Medicine, National Yang Ming University, Taipei, Taiwan

**Keywords:** Non-alcoholic fatty liver disease, Steatohepatitis, Statins, Fibrogenesis, Hepatocyte, Paracrine effect

## Abstract

**Background:**

Non-alcoholic steatohepatitis (NASH) is associated with hepatic fibrogenesis. Despite well-known cholesterol-lowering action of statins, their mechanisms against NASH-mediated fibrogenesis remain unclear. This study aimed at investigating the *in vitro* and *in vivo* anti-fibrotic properties of fluvastatin (Flu).

**Methods:**

Palmitate (PA)-induced changes in intracellular hydrogen peroxide levels in primary rat hepatocytes (PRHs) and human hepatoma cell line (HepG2) were quantified by dichlorofluorescein diacetate (DCF-DA) dye assay, whereas changes in expressions of NADPH oxidase gp91^*phox*^ subunit, α-smooth muscle actin (α-SMA), and NFκB p65 nuclear translocation were quantified with Western blotting. Quantitative real-time polymerase chain reaction (q-PCR) was used to investigate mRNA expressions of pro-inflammatory genes (ICAM-1, IL-6, TNF-α). Conditioned medium (CM) from PA-treated PRHs was applied to cultured rat hepatic stellate cell line, HSC-T6, with or without Flu-pretreatment for 2 h. Pro-fibrogenic gene expressions (COL1, TIMP-1, TGF-β1, α-SMA) and protein expression of α-SMA were analyzed. *In vivo* study using choline-deficient L-amino acid defined (CDAA) diet-induced rat NASH model was performed by randomly assigning Wistar rats (n = 28) to normal controls (n = 4), CDAA diet with vehicles, and CDAA diet with Flu (5 mg/kg or 10 mg/kg) (n = 8 each) through gavage for 4 or 8 weeks. Livers were harvested for histological, Western blot (α-SMA), and q-PCR analyses for expressions of pro-inflammatory (IL-6, iNOS, ICAM-1) and pro-fibrogenic (Col1, α-SMA, TIMP-1) genes.

**Results:**

*In vitro*, Flu (1–20 μM) inhibited PA-induced free-radical production, gp91^*phox*^ expression, and NFκB p65 translocation in HepG2 and PRHs, while CM-induced α-SMA protein expression and pro-fibrogenic gene expressions in HSC-T6 were suppressed in Flu-pretreated cells compared to those without pretreatment. Moreover, α-SMA protein expression was significantly decreased in HSC-T6 cultured with CM from PA-Flu-treated PRHs compared to those cultured with CM from PA-treated PRHs. Flu also reduced steatosis and fibrosis scores, α-SMA protein expression, mRNA expression of pro-inflammatory and pro-fibrogenic genes in livers of CDAA rats.

**Conclusions:**

We demonstrated PA-induced HSC activation through paracrine effect of hepatocyte *in vitro* that was significantly suppressed by pre-treating HSC with Flu. *In vivo*, Flu alleviated steatosis-induced HSC activation and hepatic fibrogenesis through mitigating inflammation and oxidative stress, suggesting possible therapeutic role of Flu against NASH.

## Background

Non-alcoholic fatty liver disease (NAFLD) has become a public health issue because of the ongoing epidemics of obesity and type 2 diabetes. The spectrum of NAFLD ranges from simple steatosis to steatohepatitis, liver fibrosis, cirrhosis, and eventually hepatocellular carcinoma [1]. According to the “two-hit-hypothesis” of non-alcoholic steatohepatitis (NASH), hepatic steatosis (“first hit”) is a prerequisite for the development of subsequent adverse events (“second hit”) when combined with unfavorable environmental and genetic factors, leading to inflammatory liver damage and fibrogenesis in NASH [2]. In humans with NAFLD, circulating free fatty acids (FFAs) are commonly elevated and their plasma levels correlate with disease severity. Consistently, FFAs have been proposed to contribute to the development and progression of NAFLD and NASH [3,4].

NASH is known to be associated with an increased generation of reactive oxygen species [5] through nicotinamide adenine dinucleotide phosphate (NADPH) oxidase-mediated pathways that play a crucial role in the development of hepatic fibrogenesis [6]. On the other hand, nuclear factor kappa (NFκB) is a dimeric transcription factor comprised of five family members RelA (p65), RelB, c-Rel, p50 and p52 and is a critical mediator of inflammatory responses [7,8]. Recent studies have shown that modulation of lipid homeostasis and suppression of NFκB activation counteract the progression of liver injury in experimental NASH model [9].

Statins are specific inhibitors of 3-hydroxy-3methylglutaryl coenzyme A (HMG-CoA) reductase, the rate-limiting enzyme in cholesterol biosynthesis [10]. In addition to their cholesterol-lowering effects, statins also possess pleiotropic properties that account for their anti-inflammatory, anti-proliferative, anti-thrombotic, anti-oxidative, anti-cancer, and immunomodulatory actions *in vitro* and *in vivo*. It has been shown that statins can reduce liver triglyceride [11] and ameliorate severe hepatic steatosis [12], thereby improving NASH-related fibrogenesis. Clinically, the therapeutic role of statins in the treatment of patients with NASH is still controversial [13]. Although it has been reported that statins could improve liver steatosis and reduce the NAFLD activity score [14], their efficacy against fibrosis remains unclear. This study, therefore, aimed at investigating the therapeutic potential of statins against NASH-related fibrogenesis through *in vitro* and *in vivo* studies.

## Methods

### Cell culture

A human hepatoma cell line, HepG2, was grown in Dulbecco’s modified Eagle’s medium (DMEM) with 10% heat-inactivated fetal bovine serum (FBS). Hepatocytes for primary culture were isolated from male Wistar rats (275–300 g) using a two-stage non-recirculating perfusion method [15]. Primary rat hepatocytes (PRHs) were collected and cultured in 6-well plates coated with collagen film (Vitrogen 100; Collagen Corp., Palo Alto, CA). Conditioned medium (CM) was collected from hepatocytes cultured with 0.1% FBS to minimize confounding effects from factors present in serum [16]. The hepatic stellate cell (HSC)‐T6 cell line, an immortalized rat cell line of HSCs from Professor S. L. Friedman of the Mount Sinai School of Medicine (New York, USA) as a generous gift, were maintained in DMEM (containing 10% FBS). Bioassay systems for the HSC‐T6 cell line [17] have been established in our laboratory [18,19].

### Chemicals and antibodies

Sodium palmitate (PA) and bovine serum albumin (BSA) were purchased from Sigma-Aldrich (St. Louis, MO, USA). PA stock solution was prepared as previously described [20]. Briefly, a 100-mM PA stock solution was prepared in DMSO. A filtered 3% (w/v) FFA-free BSA solution was prepared with ddH_2_O and maintained at 55°C in a water bath. Then 10 μM FFA/BSA solution was obtained by complexing the appropriate amount of PA stock solution with 3% BSA at 55°C for another 30 min. The above solution was freshly prepared before use. Fluvastatin (Flu) used in this study (Lescol®, fluvastatin sodium) was kindly provided by Novartis company. Anti‐α-SMA, α-tubulin, PCNA, and NFκB (p65) antibodies were purchased from Santa Cruz (Santa Cruz, CA, USA), while anti-NADPH oxidase gp91^*phox*^ subunit and β-catenin antibodies were from Millipore (Bedford, MA, USA).

### Measurement of reactive oxygen species (ROS) production

Intracellular H_2_O_2_ levels were measured by the conversion of non-fluorescent 2′,7′-dichlorofluorescein diacetate (DCF-DA) into 2′,7′-dichlorofluorescein (DCF) [15]. Cells were incubated with 8-μM DCF-DA for 30 min at 37°C before treatment with PA. The fluorometric analysis was performed at indicated time points by the excitation and emission wavelengths at 485 nm and 530 nm, respectively.

### Cytotoxicity assay

The assay of the reduction of 3‐ (4,5‐dimethylthiazol‐2‐yl)‐2,5‐ diphenyltetrazolium bromide (MTT) was used to evaluate the potential of PA cytotoxicity. PRHs or HepG2 cells were incubated in 24‐well plates (1 × 10^5^ cells/well) containing DMEM medium with or without different concentrations of PA and fluvastatin (Flu) for 24 h. The absorption intensity at 540 nm was measured by an ELISA reader. The relative cell viability was determined by the amount of MTT converted to the insoluble formazan salt. The optical density of the formazan formed in the control cells was taken as 100% viability.

### Western blotting analysis

PRHs or HepG2 cells cultured in the presence or absence of PA or Flu for indicated time points were collected for protein analyses. Expression of NADPH oxidase (NOX) subunit gp91^*phox*^ in membrane fractions was assessed with subcellular fractionation. Cytoplasmic and nuclear extracts were prepared using a nuclear extraction kit (Chemicon, Temecula, CA) according to the manufacturer’s instructions and our published methods [18,19]. HSC-T6 cells were pretreated with or without Flu (1 and 5 μM) for 2 h before being incubated with a conditioned medium (CM) collected from PRHs treated with PA for 24 h to study the protein expression of α‐SMA. In addition, CM from PRHs treated with or without Flu was also collected and was added to HSC-T6 cells to study the protein expression of α‐SMA. Protein lysates from total cell, cytoplasm, membrane or nuclear fraction were separated with SDS-PAGE and transferred onto Immobilon‐PVDF (Millipore, Bedford, MA, USA). After 1-h blocking for nonspecific binding, blots were incubated with specific primary antibodies (1:10000 dilution) against α‐actin (GTX100095, GeneTex), α‐tubulin (sc-8035, Santa Cruz Biotechnology, Santa Cruz, CA, USA), PCNA (sc-56, Santa Cruz), NFκB (p65) (MAB3026, Santa Cruz), gp91^*phox*^ (07–024, Millipore) or β-catenin (06–734, Millipore). After 1-h incubation with HRP-conjugated secondary antibodies (1:2000) (Santa Cruz), immunodetection was performed by the Luminescence Imaging System (FUJI las-4000mini, Japan).

### Reverse transcription and quantitative polymerase chain reaction (q-PCR)

Total RNA was extracted from liver tissue, cultured PRHs, HepG2 or HSC-T6 using the Tri-reagent (Sigma-Aldrich, St. Louis, MO, USA) according to the protocol provided by the manufacturer. One microgram of RNA was reversely transcribed to cDNA by using RevertAidTM First Strand cDNA Synthesis kit (Fermentas, Burlington, CA, USA). The mRNA levels were analyzed in triplicates with the use of Smart Quant Green Master mix (Protech, Taiwan) by LightCycler 480 Real-Time PCR System (Roche, Indianapolis, IN, USA) and normalized to GAPDH mRNA expression. The sequences of the primers used for quantitative PCR were listed in Table 1.

**Table 1:** **Primer sequences for quantitative PCR**

Species	Gene	Forward sequence (5′-3′)	Reverse sequence (5′-3′)
Rat	GAPDH	CAAGGTCATCCTGACAACTTTG	GTCCACCACCCTGTTGCTGTAG
	ICAM1	TCATGCCCGTGAAATTATGGT	AGGACCCTTCTAAGTGGTTGGAA
	IL6	TTCCTACCCCAACTTCCAATG	ATGAGTTGGATGGTCTTGGTC
	TNFα	GACCCTCACACTCAGATCATCTTCT	TGCTACGACGTGGGCTACG
	SMA	TTCGTTACTACTGCTGAGCGTGAGA	AAAGATGGCTGGAAGAGGGTC
	TGFβ1	TATAGCAACAATTCCTGGCG	TGCTGTCACAGGAGCAGTG
	TIMP1	GCCTACACCCCAGCCAT	ATGCCAGGGAACCAGGAAGC
	Col1	CAATGGCACGGCTGTGTGCG	CACTCGCCCTCCCGTCTTTGG
	iNOS	CAGCCCTCAGAGTACAACGAT	CAGCAGGCACACGCAATGAT
Human	GAPDH	GCCAAAAGGGTCATCATCTC	GGCCATCCACAGTCTTCT
	ICAM1	ATGCCCAGACATCTGTGTCC	GGGGTCTCTATGCCCAACAA
	IL6	GGTACATCCTCGACGGCATCT	GTGCCTCTTTGCTGCTTTCAC
	TNFα	CCCAGGGACCTCTCTCTAATC	ATGGGCTACAGGCTTGTCACT

### Animal model with hepatic steatosis and fibrosis

A variety of animal models for NAFLD/NASH have been reported to date, including those of genetically predisposed, diet-induced, and a combination of the two. Choline-deficient L-amino acid defined (CDAA) diet was chosen in this study to investigate the anti-fibrotic role of statin. In this model, due to the lack of oligopeptides consumption, severe methyl-deficient state develops with concomitant lowering of antioxidant defenses, contributing to its fibrogenicity and carcinogenicity [21]. The advantage of choline-deficient L-amino acid defined (CDAA) diet model is the rapid hepatic accumulation of lipids and reliable development of fibrosis [22].

Adult male Wistar rats (BioLASCO Taiwan), weighing 180–200 g, were randomly divided into four groups. (i) Normal controls: Rats receiving regular animal chow (n = 8); (ii) CDAA: Rats receiving CDAA diet (n = 16) (no. 518753, Dyets) [21]; (iii) CDAA rats receiving Flu 5 mg/kg/day (n = 16), and (iv) CDAA rats receiving Flu 10 mg/kg/day (n = 16), each given by daily gavages. Half of the animals from each group were sacrificed after 4 weeks of Flu treatment, while the rest were sacrificed after 8 weeks of treatment. The whole liver was taken and the left lobe was sampled for subsequent experiments. All animal experimental procedures were approved by the Institutional Animal Care and Use Committee (IACUC) of National Yang Ming University (Approval No. 981266) and National Research Institute of Chinese Medicine (Approval No.102-350-1), in accordance with the Guide for the Care and Use of Laboratory Animals (National Academic Press, USA, 1996). Serum alanine transaminase (ALT) and aspartate transaminase (AST), creatinine (Cr), cholesterol (Cho), triglyceride (TG), and glucose (Glu) levels were measured following standard procedures using a colorimetric analyzer (Dri‐Chem 3000). Serum TNF‐α concentrations were determined by specific and sensitive enzyme linked immunoassays (R &D Systems) [23].

### Histopathological examination

Formalin-fixed, paraffin-embedded liver sections were deparaffinized and stained with hematoxylin-eosin and Sirius red for histological examination and determination of collagen distribution, respectively [24]. The scoring system used in the present study was adapted from that previously reported by Brunt *et al.* that histologically assesses the extent of steatosis, hepatocyte ballooning, inflammation, and fibrosis [25] as well as the NAFLD activity score (NAS) designed by the NASH Clinical Research Network(CRN) based on the summation of the scores of three key parameters of NASH including steatosis (0–3), lobular inflammation (0–3), and ballooning (0–2) with a final score ranging from 0 to 8 [26]. Besides, fibrosis was staged as: Stage 0: No fibrosis; Stage 1: Zone 3 perisinusoidal/pericellular fibrosis, focally or extensively present; Stage 2: Zone 3 perisinusoidal/pericellular fibrosis with focal or extensive periportal fibrosis; Stage 3: Zone 3 perisinusoidal/pericellular fibrosis and portal fibrosis with focal or extensive bridging fibrosis; Stage 4: Cirrhosis. The scores were given by a pathologist (Y.‐T. C.) blinded to the rats’ treatment assignment after examination of three different areas of a liver section for each rat.

### Statistical analysis

Data are expressed as the mean ± SEM. One‐way analysis of variance (ANOVA) was used for comparison of biochemical and molecular parameters. A non‐parametric method (the Dunn procedure under the Kruskal–Wallis test) was used for multiple pairwise comparisons between groups for analysis of the histological grades of fibrosis. Statistical significance was accepted at p < 0.05.

## Results

### Flu attenuates PA-induced oxidative stress, inflammatory responses, and fibrogenesis

#### Inhibition of ROS production and NADPH oxidase gp91^phox^ expression in PA-treated HepG2 and PRHs by Flu

**Figure 1:**
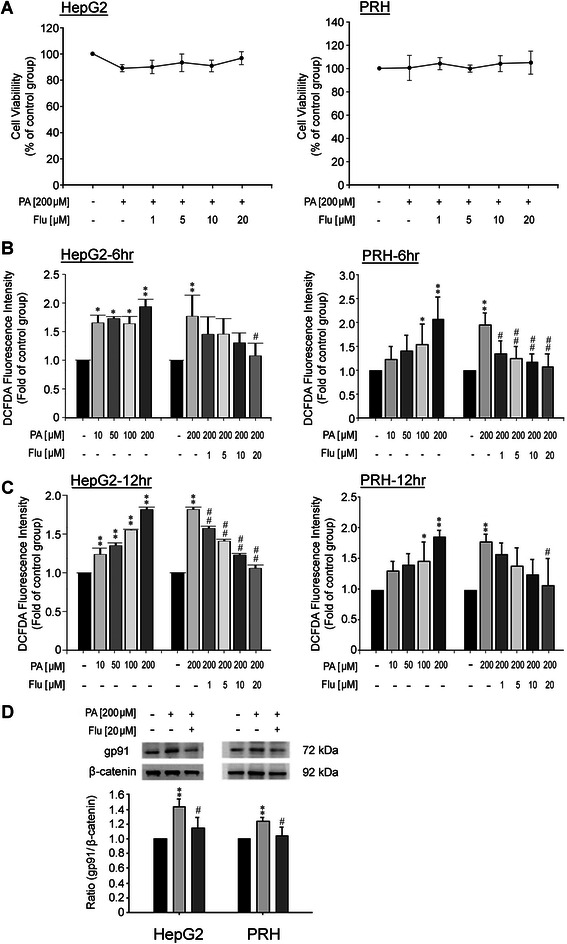
**Effects of fluvastatin (Flu) on cell viability, ROS production and NADPH oxidase subunit gp91**^***phox***^**expression in HepG2 cells and primary rat hepatocytes (PRHs). (A)** Effects of Flu on cell viability of HepG2 cells and PRHs at 24 hr after treatment (n = 3). **(B)** Flu significantly reduced the reactive oxygen species (ROS) production of PA-treated HepG2 cells and PRHs at 6 hr after treatments. ^*^p < 0.05 and ^**^p < 0.01 *vs*. control; ^#^p < 0.05 and ^##^p < 0.01 *vs*. PA treatment (n = 3). **(C)** Flu significantly attenuated the ROS production of PA-treated HepG2 and PRHs at 12 hr after treatments. ^*^p < 0.05 and ^**^p < 0.01 *vs*. control; ^#^p < 0.05 and ^##^p < 0.01 *vs*. PA treatment (n = 3). **(D)** Flu significantly decreased the expression of NADPH oxidase gp91^*phox*^ in PA-treated HepG2 cells (left panel) and PRHs (right panel). ^**^p < 0.01 *vs*. control; ^#^p < 0.05 *vs*. PA treatment (n = 3).

To demonstrate the anti-fibrotic roles of Flu in steatosis-induced liver fibrosis, the beneficial effects of Flu on lipid-induced hepatocyte damage were first investigated. PRHs (isolated from Wistar rats) and HepG2 cells were cultured in the presence of PA, the most abundant free fatty acid in circulation. Firstly, we demonstrated that PA and Flu treatment did not alter the cell viability of PRHs and HepG2 cells after 24 hours of exposure (Figure 1A). PA treatment significantly enhanced ROS production in both HepG2 cells and PRHs at 6 hour and 12 hour detected by fluorescence DCF-DA dye (Figure 1B and C). PA (200 μM) significantly stimulated ROS production, and Flu (1–20 μM) concentration-dependently reduced PA-induced ROS production (Figure 1B and C), with higher concentrations achieving significant reduction in PRHs at 12 hour (Figure 1C). PA (200 μM) also increased gp91^*phox*^ expression in HepG2 and PRHs (Figure 1D). By contrast, Flu (20 μM) reduced gp91^*phox*^ expression in both cell types, indicating suppression of ROS generation after Flu treatment (Figure 1D).

#### Suppression of NFκB p65 nuclear translocation and pro-inflammatory gene transcripts in PA-treated HepG2 and PRHs by Flu

**Figure 2:**
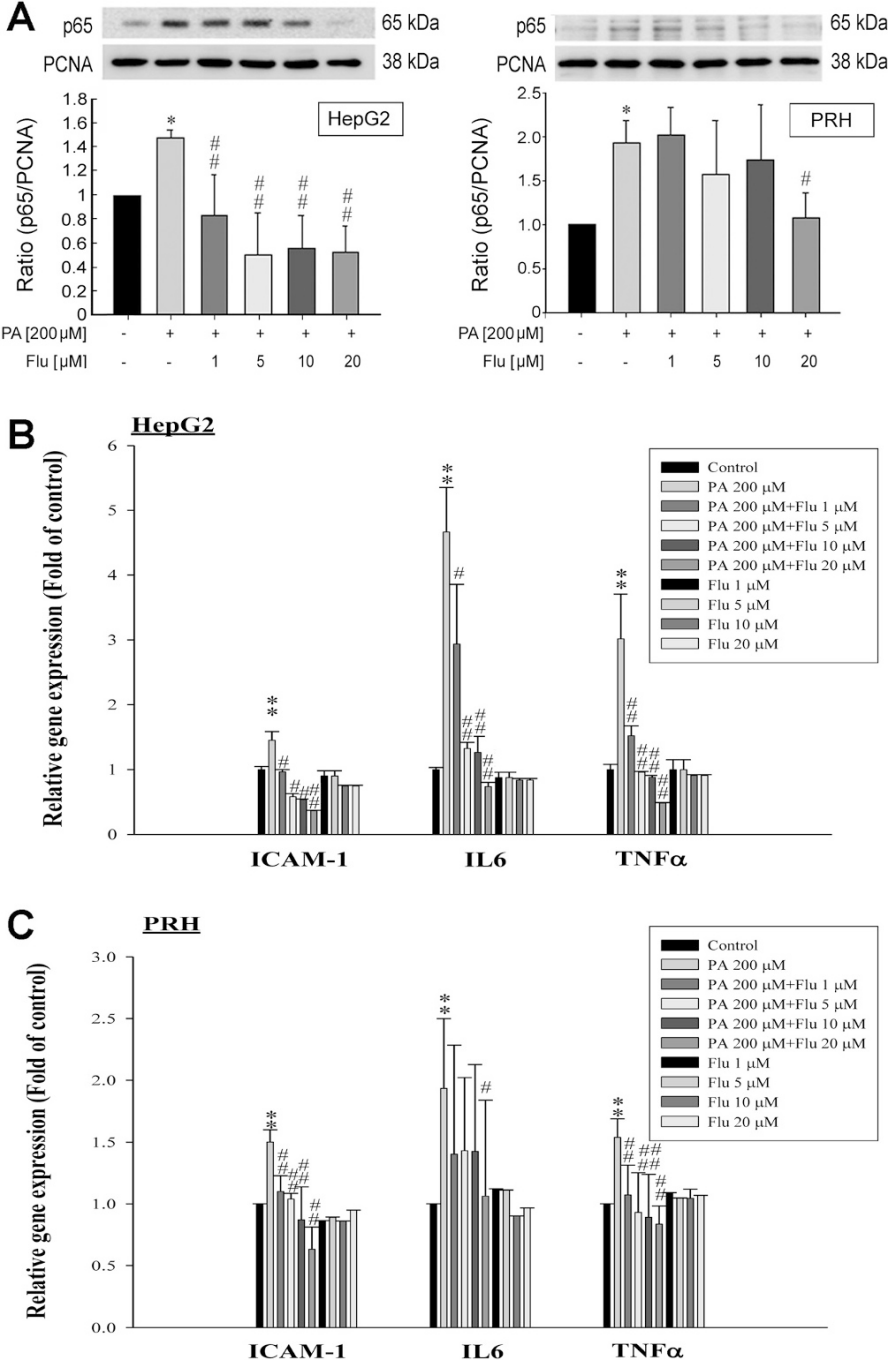
**Effects of fluvastatin (Flu) on NFκB p65 nuclear translocation, mRNA expression levels of pro-inflammatory genes in HepG2 cells and primary rat hepatocytes (PRHs). (A)** Pre-treatment with Flu for 2 hr reduced the NFκB p65 nuclear translocation in PA-treated HepG2 cells and PRHs at 6 hr after treatment. ^*^p < 0.05 *vs*. control; ^#^p < 0.05 and ^##^p < 0.01 *vs*. PA treatment (n = 3). **(B)** Flu treatment inhibited the mRNA expressions of ICAM-1, IL-6 and TNF-α of PA-treated HepG2 cells while there were no significant differences in the expressions of ICAM-1, IL-6 and TNF-α between the control group and the group treated with Fluvastatin alone. ^**^p < 0.01 *vs*. control; ^#^p < 0.05 and ^##^p < 0.01 *vs*. PA treatment (n = 3). **(C)** Flu treatment decreased the mRNA expressions of ICAM-1, IL-6 and TNF-α of PA-treated PRHs, but no significant differences in the expressions of these markers between the control group and the group treated with Fluvastatin alone were noted. ^**^p < 0.01 *vs*. control; ^#^p < 0.05 and ^##^p < 0.01 *vs*. PA treatment (n = 3).

While PA (200 μM) stimulated NFκB p65 nuclear translocation in both HepG2 and PRHs (Figure 2A), Flu (1–20 μM) attenuated NFκB p65 nuclear translocation in both cell types (Figure 2A). In addition, Flu treatment inhibited the mRNA expression levels of pro-inflammatory gene transcripts (ICAM-1, IL-6, TNF-α) in both PA-treated HepG2 cells and PRHs (Figure 2B and C). Moreover, there were no significant differences in the expressions of pro-inflammatory gene transcripts between the control group and the group treated with Flu alone (Figure 2B and C).

#### Suppression of α-SMA expression and pro-fibrogenic gene transcripts in CM or TGF-β1 -treated HSC-T6 cells by Flu

**Figure 3:**
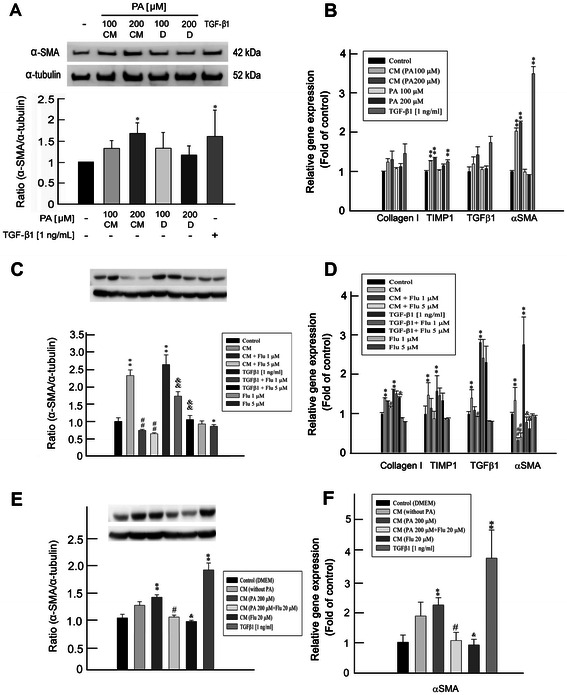
**Effects of fluvastatin (Flu) on α-SMA protein expression and pro-fibrogenic gene transcripts in conditioned medium (CM) or TGF-β1-treated HSC-T6 cells. (A)** HSC-T6 cells incubated in the conditioned medium (CM) from PA-treated PRHs showed significantly increased α-SMA protein expression, while α-SMA protein expression was not up-regulated in HSC-T6 cells treated directly with PA. TGF-β1 (1 ng/mL) was used as a positive control ^*^p < 0.05 *vs*. control. **(B)** mRNA expressions of TIMP-1 and α-SMA were increased in HSC-T6 cells incubated with CM, whereas there were no significant differences in the expressions of pro-fibrogenic genes when HSC-T6 cells were treated with PA directly. TGF-β1 (1 ng/mL) was used as a positive control. ^**^p < 0.01 *vs*. control. **(C)** Treatment with Flu significantly reduced CM- and TGF-β1-induced α-SMA protein expression. Besides, α-SMA protein expression was also suppressed in HSC-T6 cells incubated with Flu (5 μM) alone. ^*^p < 0.05 *vs*. control; ^**^p < 0.01 *vs*. control; ^##^p < 0.01 *vs*. CM treatment; &&p < 0.01 *vs*. TGF-β1 treatment. **(D)** Flu treatment attenuated CM-induced mRNA expressions of Col1, TGF-β1 and α-SMA, and TGF-β1-induced mRNA expressions of Col1 and α-SMA. ^**^p < 0.01 *vs*. control; ^#^p < 0.05 *vs*. CM treatment; ^##^p < 0.01 *vs*. CM treatment; &p < 0.05 *vs*. TGF-β1 treatment. **(E & F)** α-SMA protein and mRNA expressions were significantly decreased in HSC-T6 cells incubated with CM collected from PA-Flu-treated PRHs compared to those incubated with CM collected from PA-treated PRHs. Furthermore, α-SMA protein and mRNA expressions were also reduced in HSC-T6 cells when treated with CM collected from Flu-treated PRHs compared to those incubated with CM without Flu treatment. ^**^p < 0.01 *vs*. control; ^#^p < 0.05 *vs*. CM (PA 200 μM) treatment; &p < 0.05 *vs*. CM without Flu treatment. n=3 for all experiments.

To test if the activation of HSC was induced directly by PA or indirectly through the paracrine effect of hepatocytes, we cultured HSC-T6 cells either (a) in the presence of conditioned medium (CM) from PA-treated PRHs or (b) directly with PA. Transforming growth factor (TGF)-β1 (1 ng/mL) was used as a positive control to stimulate α-SMA expression and pro-fibrogenic gene transcription [18]. Figure 3A showed a significant increase of α-SMA protein expression in HSC-T6 cells incubated with CM from PA (200 μM)-treated PRHs, whereas PA did not significantly induce α-SMA expression in HSC-T6 cells directly both at 100 and 200 μM. In addition, mRNA expressions of TIMP-1 and α-SMA were increased in HSC-T6 cells incubated with CM from PA-treated PRHs, whereas there was no significant difference in the expressions of pro-fibrogenic genes when HSC-T6 cells treated directly with PA (Figure 3B). These results indicate that, instead of direct activation of HSCs by PA, HSCs were indirectly activated by PA via PA-elicited paracrine effect of hepatocytes. Moreover, treatment with Flu significantly reduced CM- and TGF-β1-induced α-SMA expression in cultured HSCs (Figure 3C). Additionally, α-SMA protein expression was also suppressed in HSC-T6 cells incubated with Flu (5 μM) alone, indicating that Flu can inhibits activation of HSCs directly. Figure 3D showed a significant increase of mRNA expressions of pro-fibrogenic genes (Col1, TIMP-1, TGF-β1, and α-SMA) in HSC-T6 cells incubated with CM and TGF-β1 (1 ng/ml). Flu treatment attenuated CM-induced mRNA expressions of Col1, TGF-β1 and α-SMA, and TGF-β1-induced mRNA expressions of Col1 and α-SMA (Figure 3D).

#### Suppression of α-SMA expression in HSC-T6 cells by CM from Flu-treated PRHs

To further investigate the effects of Flu on upstream mechanism of HSC activation, we stimulated the HSCs with the CM from PRHs with or without PA treatment, and CM from PRHs with or without Flu treatment. Figure 3E and F showed that both α-SMA mRNA and protein expressions were significantly reduced in HSCs incubated with CM collected from PA-Flu-treated PRHs compared to those incubated with CM collected from PA-treated PRHs. Furthermore, α-SMA mRNA and protein expressions were also decreased in HSC-T6 when treated with CM collected from Flu-treated PRHs compared to those incubated with CM without Flu treatment (Figure 3E and F). These results indicate that Flu not only inhibited α-SMA expression directly in HSC-T6 (Figure 3C), but it also decreased α-SMA expression in HSC-T6 cells incubated with CM collected from Flu-treated PRHs.

### Anti-fibrotic effects of Flu in CDAA rats

#### Changes in general features, expressions of serum biomarkers, and histology

To further evaluate the potential anti-fibrotic role of Flu, a CDAA diet-induced NASH animal model was used. The effects of Flu on the general features of CDAA rats were summarized in Table 2. Four-week and 8-week CDAA rats showed remarkably higher plasma AST (503 ± 89 *vs.* 96 ± 13 U/mL, *p* < 0.05; 373 ± 30 *vs.* 77 ± 4 U/mL, *p* < 0.01) and ALT (134 ± 28 *vs.* 25 ± 2 U/mL, *p* < 0.05; 161 ± 16 *vs.* 25 ± 5 U/mL, *p* < 0.01) levels compared to those in the normal controls, indicating hepatic injury in the formers (Table 2). Levels of ALT in CDAA rats after 8-week Flu treatment were significantly decreased after receiving high-dose (i.e., 10 mg/kg/day) Flu compared to those without Flu treatment (84 ± 24 *vs.* 161 ± 16 U/mL, *p* < 0.01). Moreover, as shown in Table 2, Flu treatments (5 mg/kg and 10 mg/kg/day) for either 4 or 8 weeks significantly attenuated plasma TNF-α level in CDAA rats compared to that in the untreated CDAA group (4–week: 67 ± 11 pg/mL and 64 ± 9 pg/mL vs. 109 ± 12 pg/mL for doses of 5 mg/kg and 10 mg/kg/day, respectively, both *p* < 0.01; 8–week: 30 ± 11 pg/mL vs. 55 ± 22 pg/mL, *p* < 0.01; 11 ± 3 pg/mL vs. 55 ± 22 pg/mL, *p* < 0.05 for doses of 5 mg/kg and 10 mg/kg/day, respectively).

**Table 2:** **General features and serum biochemical analyses in rats fed with choline-deficient L-amino acid defined (CDAA) diet receiving fluvastatin (Flu) or vehicle treatment compared with normal controls**

Groups	4 wk				8 wk			
	Control	CDAA	CDAA + Flu (5 mg/kg)	CDAA + Flu (10 mg/kg)	Control	CDAA	CDAA + Flu (5 mg/kg)	CDAA + Flu (10 mg/kg)
**Numbers**	4	8	8	8	4	8	8	8
**Steatosis score**	0.00 ± 0.00	3.00 ± 0.00 ^**^	3.00 ± 0.00 ^**^	2.70 ± 0.95 ^**^	0.00 ± 0.00	3.00 ± 0.00 ^**^	3.00 ± 0.00 ^**^	2.25 ± 1.17 ^#^
**Hepatocyte ballooning**	0.0 ± 0.0	2.0 ± 0.0 ^**^	2.0 ± 0.0 ^**^	2.0 ± 0.0 ^**^	0.0 ± 0.0	2.0 ± 0.0 ^**^	2.0 ± 0.0 ^**^	1.5 ± 0.8 ^**^
**Inflammation**	0.00 ± 0.00	0.60 ± 0.52	0.80 ± 0.42 ^**^	0.40 ± 0.52	0.00 ± 0.00	1.17 ± 0.25 ^**^	1.60 ± 0.39 ^**^	1.0 ± 0.55 ^**,†^
**Fibrosis score**	0.00 ± 0.00	1.80 ± 0.42 ^**^	1.20 ± 0.42 ^# #^	0.70 ± 0.48^# #, †^	0.00 ± 0.00	1.67 ± 0.56 ^**^	1.15 ± 0.63 ^**^	0.58 ± 0.92 ^#^
**BW (g)**	312 ± 7	245 ± 9 ^**^	217 ± 6 ^**^	195 ± 8 ^**^^##^	467 ± 10	442 ± 12	419 ± 7 ^**^	399 ± 7 ^**^^#^
**LW (g)**	10.9 ± 0.4	18.1 ± 0.9 ^**^	14.7 ± 0.9 ^** #^	14.3 ± 0.4 ^**^^##^	14.3 ± 0.6	23.5 ± 0.9 ^**^	20.3 ± 0.7 ^**^	18.2 ± 1.8 ^##^
**AST (U/L)**	96 ± 13	503 ± 89 ^*^	439 ± 90 ^*^	366 ± 72	77 ± 4	373 ± 30 ^**^	311 ± 26 ^**^	267 ± 49 ^**^
**ALT (U/L)**	25 ± 2	134 ± 28 ^*^	160 ± 25 ^**^	121 ± 20	25 ± 5	161 ± 16 ^**^	125 ± 11 ^**^	84 ± 24 ^##^
**Cr (mg/dL)**	0.43 ± 0.02	0.51 ± 0.05	0.48 ± 0.03	0.47 ± 0.04	0.40 ± 0.00	0.42 ± 0.01	0.44 ± 0.02	0.41 ± 0.01
**TG (mg/dL)**	47.5 ± 5.3	22.9 ± 1.9 ^**^	26.4 ± 2.79 ^**^	29.7 ± 4.7 ^*^	58 ± 9	30 ± 4 ^*^	24 ± 3 ^*^	41 ± 7
**Cholesterol (mg/dL)**	69.0 ± 4.4	56.4 ± 1.9	56.3 ± 2.3	60.7 ± 4.4	63 ± 6	55 ± 2	57 ± 2	58 ± 4
**Glucose (mg/dL)**	158 ± 9	124 ± 6	132 ± 11	126 ± 10	134 ± 12	123 ± 5	127 ± 6	132 ± 3
**TNFα (pg/mL)**	50 ± 4	109 ± 12 ^**^	67 ± 11 ^##^	64 ± 9 ^##^	35 ± 7	55 ± 22 ^*^	30 ± 11 ^##^	11 ± 3 ^#^

*p < 0.05, **p < 0.01 *vs.* control group, ^#^*p* < 0.05, ^##^*p* < 0.01 *vs.* CDAA group, †p < 0.05 vs. CDAA + Flu (5 mg/kg). BW: Body weight, LW: Liver weight, AST: Aspartate aminotransferase, ALT: Alanine aminotransferase, Cr: Creatinine, TG: Triglyceride, TNFα: Tumor necrosis factor α.

**Figure 4:**
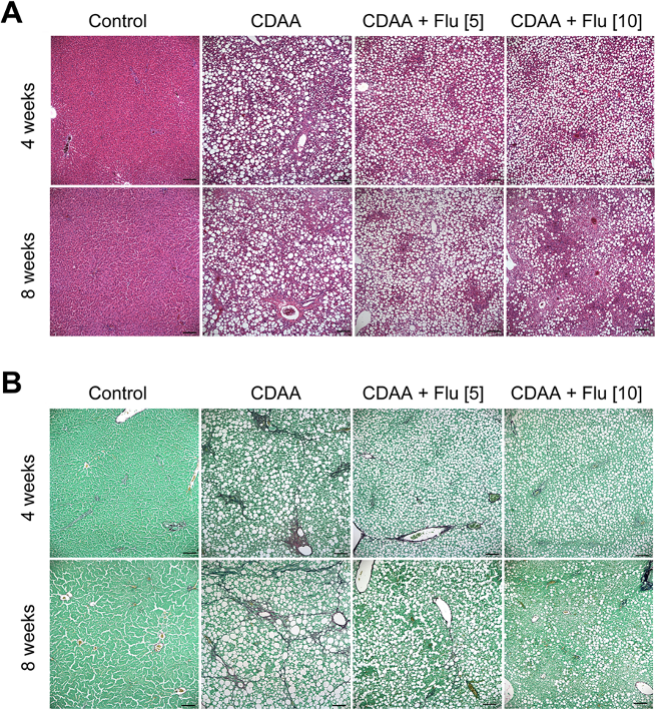
**Histological examination of liver sections.** Representative liver sections were obtained from rats of normal controls (n = 4), rats fed with CDAA diet with vehicles, and rats fed with CDAA diet with Flu (5 mg/kg or 10 mg/kg) (n = 8 each). Sections were stained with **(A)** Hematoxylin-eosin or **(B)** Sirius red. Scale bar = 100 μm.

Histological examination of the livers from rats consuming CDAA diet for 4 and 8 weeks revealed progressive lipid accumulation, formation of fibrous septa, and loss of hepatocytes compared with those from normal controls (Figure 4A). Sirius Red staining of the liver indicated distinct deposits of collagen fibers after 4-week and 8-week feeding with CDAA diet (Figure 4B). Flu treatment (5 mg/kg/day and 10 mg/kg/day) attenuated the deposition of collagen fibers (Figure 4B). Steatosis scores of the livers from 8-week high-dose Flu-treated CDAA rats (2.25 ± 1.17) were significantly lower than that in untreated CDAA rats (3.00 ± 0.00, *p* < 0.05, Table 2), whereas there was no notable difference between CDAA rats receiving 4-week high-dose Flu and their untreated counterparts. Besides, no significant reduction in steatosis score was noted between the low-dose Flu treatment group and the untreated CDAA animals after either 4-week or 8-week treatment. Treating CDAA animals with either low- or high-dose Flu for 4 weeks significantly suppressed the elevations in fibrosis score compared to that of untreated CDAA rats (both *p* < 0.01), whereas the reduction in fibrotic score was significant only in CDAA rats receiving 8-week-high-dose Flu treatment (*p* < 0.05). Furthermore, there were significant differences in the degree of fibrosis in rats being given different dosages at the 4th week but not at 8th week (Table 2).

#### Protein expression of α-SMA in the livers of CDAA rats

**Figure 5:**
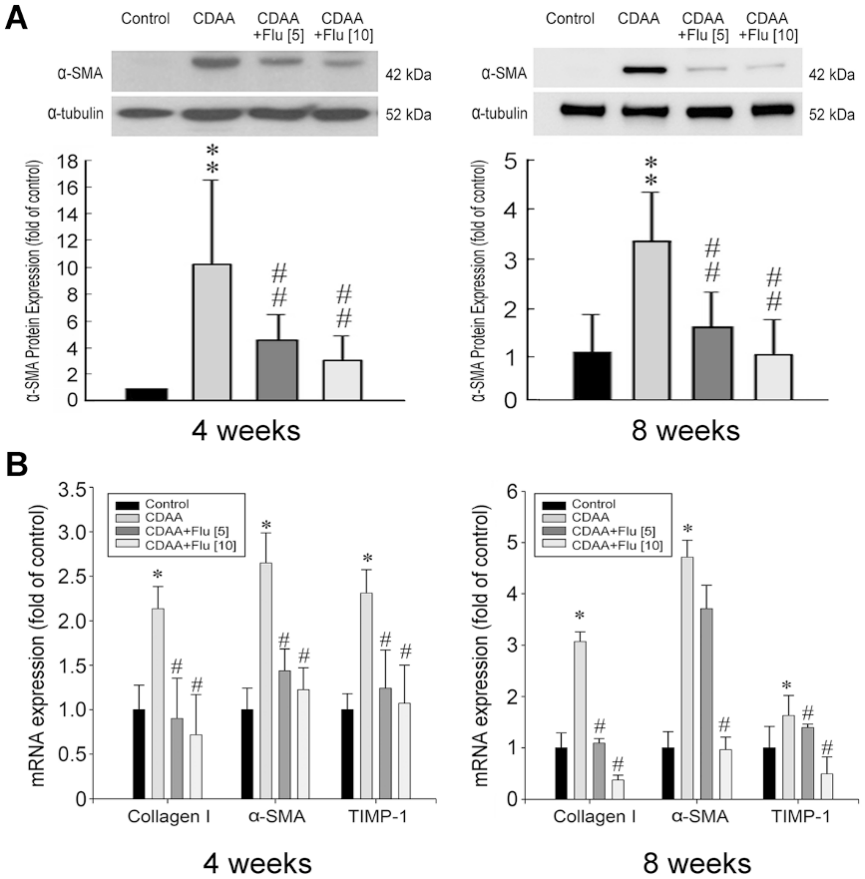
**Anti-fibrotic effects of fluvastatin (Flu) on CDAA rats. (A)** Representative result showed that Flu treatment reduced the protein expression of α-SMA in the liver tissues in CDAA rats at 4 and 8 weeks. ^**^p < 0.01 *vs*. control rat; ^##^p < 0.01 *vs*. CDAA rat. **(B)** Quantitative PCR analyses for the expressions of Collagen I, α-SMA, and TIMP-1 transcripts in control rats, CDAA rats and CDAA rats receiving Flu (5 or 10 mg/kg/day). Densities of Collagen I, α-SMA, and TIMP-1 to G3PDH mRNA levels were analyzed by computerized densitometry and are expressed as the indicated ratios, respectively. Flu treatment attenuated the expressions of these pro-fibrogenic genes. The number of rats in each column was 8. ^*^p <0.05 *vs*. control rat; ^#^p < 0.05 *vs*. CDAA rat.

Figure 5A shows that α-SMA protein expression was increased significantly in the livers of CDAA rats compared with that in the normal controls (*p* < 0.01). Treatment with either 5 mg/kg/day or 10 mg/kg/day of Flu for 4 and 8 weeks markedly reduced α-SMA protein expression (*p* < 0.01).

#### Analyses of transcripts of pro-fibrogenic genes (Collagen 1, α-SMA, TIMP-1) and pro-inflammatory genes (IL6, iNOS and ICAM-1) in CDAA rats

**Figure 6:**
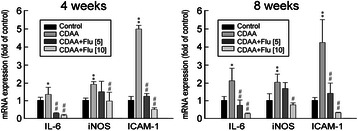
**Anti-inflammatory effects of fluvastatin (Flu) on CDAA rats.** Quantitative PCR analyses for the expressions of IL6, iNOS and ICAM-1 transcripts in control rats, CDAA rats and CDAA rats receiving Flu (5 or 10 mg/kg/day). Densities of IL6, iNOS and ICAM-1 to G3PDH mRNA levels were analyzed by computerized densitometry and are expressed as the indicated ratios, respectively. Flu treatment attenuated the expressions of these pro-inflammatory genes. The number of rats in each column was 8. ^*^p <0.05 and ^**^p <0.01 *vs*. the control rat; ^#^p < 0.05 and ^##^p < 0.01 *vs*. CDAA rat.

There were significant increases in hepatic mRNA expressions of pro-fibrogenic and pro-inflammatory genes in CDAA rats compared to those in normal controls after both 4-week and 8-week treatments (Figures 5B and 6). Additionally, Flu treatment attenuated the mRNA expressions of Collagen 1, α-SMA, TIMP-1, IL-6, iNOS, and ICAM-1 in the 4-week and 8-week Flu-treated CDAA rats (Figures 5B and 6).

## Discussion

In the present study, we demonstrated *in vitro* and *in vivo* the anti-fibrotic effects of Flu in steatosis-induced hepatocyte damage models. The results of this study showed that not only could Flu ameliorate PA-induced ROS production, NFκB activity, and pro-inflammatory gene expressions (Figures 1 and 2) in PRHs and HepG2 cells, but it could also reduce α-SMA protein expression and profibrogenic genes expressions in PA- or TGF-β1-treated HSC-T6 cells (Figure 3C and D). Furthermore, the results of the present study revealed suppression of both mRNA and protein expressions of α-SMA in HSC-T6 incubated with the CM collected from PA-Flu-treated PRHs or Flu-treated PRHs compared to those incubated with CM collected from PA-treated PRHs or CM without Flu treatment (Figure 3E and F). Consistent *in vivo* findings are the demonstration of the anti-fibrotic (Figures 4 and 5) and anti-inflammatory (Figure 6) effects of Flu in the CDAA rat model. In addition to supporting the proposal that Flu could alleviate steatosis-induced hepatic fibrogenesis through suppressing inflammation and oxidative stress, the most striking finding of the present study is the revelation of the need for paracrine effect of hepatocyte in the process of PA-elicited HSC activation.

Increased reactive oxygen species (ROS) generation has been implicated in the progression of chronic liver diseases. NOX, which is membrane-bound enzyme complex found in the plasma membrane and in the membranes of phagosomes, is present in a variety of hepatic cells including the Kupffer cells, hepatocytes, and HSCs, and participates in liver inflammation and fibrosis [27,28]. Structurally, NOX contains two membrane-integrated components, gp91^*phox*^ and p22^*phox*^, and a number of cytosolic regulatory proteins (p47^*phox*^, p67^*phox*^, p40 ^*phox*^, and Rac). The pro-fibrotic and pro-inflammatory roles of gp91^*phox*^ are illustrated by the result of a previous study that demonstrated amelioration of hepatic fibrosis through knocking out gp91^*phox*^ in mice [29,30] and that of another study showing inflammation through TNF receptor-1 expression and NFκB activation elicited by gp91^*phox*^ induction [31]. Moreover, attenuating oxidative stress and decreasing the expression of NF-κB and subsequent pro-inflammatory cytokines production have been reported to be associated with the alleviation of CCl_4_-induced hepatic fibrogenesis [32]. Our *in vitro* results demonstrated that PA induced both gp91^*phox*^ expression and NFκB p65 nuclear translocation in HepG2 and PRHs. The activation of gp91^*phox*^ and NFκB, therefore, are possible mechanisms by which lipid accumulation causes inflammation and fibrosis. Furthermore, Flu (1–20 μM) inhibited PA-induced ROS production, gp91^*phox*^ expression (Figure 1), and NFκB p65 nuclear translocation (Figure 2A), suggesting anti-oxidative, anti-inflammatory, and anti-fibrotic effects of Flu in PRHs and HepG2 cells.

A number of recent studies have reported that not only does statin reduce the concentration of circulating low-density lipoprotein cholesterol, but it also decreases hepatic lipid deposition in patients with NASH. However, it is still controversial whether it could suppress hepatic inflammation and fibrosis. In terms of organs other than the liver, several studies have previously shown that statin exerts anti-fibrotic effects on the kidneys, the lungs, and the heart [33-36]. Regarding fibrogenesis in the liver, compared to the results of the present investigation, a previous study showed that simvastatin improves NASH-related fibrosis by increasing the expression of eNOS, decreasing the expression of iNOS, and inhibiting the activation of HSC *in vitro* [37]. However, that study did not investigate the role of hepatocyte in the setting of lipidemia. In this aspect, the present study demonstrated a critical role of hepatocyte in the process of free fatty acid-induced HSC activation. *In vivo*, it has been shown that statin treatment could ameliorate the progression of hepatic steatosis, inflammation, and fibrosis induced by high-fat [37] and CDAA [38] diets, indicating that statin is protective against the pathogenesis of NASH. In this study, we consistently demonstrated the therapeutic effects of Flu on CDAA-induced liver fibrosis *in vivo* (Table 2, Figures 4 and 5), and provided the *in vitro* effects of Flu on PA-treated hepatocytes and CM-treated HSCs. Our *in vivo* results revealed the down-regulation of the mRNA expression of pro-inflammatory and pro-fibrogenic genes as well as plasma TNFα level in CDAA rats (Table 2, Figures 5 and 6) following Flu treatment, suggesting the anti-inflammatory and anti-fibrotic properties of Flu *in vivo*. On the other hand, our *in vitro* data identified increased expressions of pro-inflammatory cytokines (i.e., ICAM-1, IL-6, and TNF-α) in PA-treated hepatocytes, while Flu treatment inhibited these pro-inflammatory cytokines expressions in PA-treated hepatocytes (Figures 2B, C). Furthermore, our results also showed that α-SMA protein expression was significantly suppressed in HSC-T6 cells incubated with CM collected from PA-Flu-treated PRHs or Flu-treated PRHs compared to those incubated with CM collected from PA-treated PRHs or CM without Flu treatment (Figure 3E and F), indicating that Flu may decrease the levels of inflammatory cytokines in CM, thereby attenuating the activation of HSCs. Therefore, our results demonstrated PA-induced HSC activation through paracrine effect of hepatocyte *in vitro* that was significantly suppressed by the treatment of Flu. Accordingly, our results revealed that Flu could reduce PA-induced ROS production, gp91^*phox*^ expression (Figure 1), NFκB p65 nuclear translocation (Figure 2A), and pro-inflammatory genes expressions (Figure 2B and C) in PRHs and HepG2 cells. Consistently, Flu was found to attenuate CM-induced or TGF-β1- induced α-SMA protein expression (marker of HSC activation) and the expressions of pro-fibrogenic genes in HSC-T6 cells (Figure 3C and D). Taken together, the *in vitro* findings suggest dual therapeutic effects of Flu on both steatotic hepatocyte and HSCs.

The present study has two noteworthy limitations. Firstly, studies were not performed on animals with established diet-induced NASH to elucidate the therapeutic effects of Flu. Secondly, studies were not performed on animals treated only with Flu and regular diet to evaluate the direct effects of Flu on normal rats, its hepatotoxicity and adverse effects. Nevertheless, q-PCR on the expressions of three inflammation indicators (i.e., ICAM1, IL-6, and TNF-α) in HepG2 cells and PRHs using Flu alone were performed in the present study that showed no significant differences in the expressions of these markers between the control group and the group treated with Flu alone.

## Conclusions

In summary, we demonstrated that the anti-fibrotic effects of Flu on *in vitro* and *in vivo* NASH models could be through its anti-oxidative and anti-inflammatory properties. In addition to its well-documented lipid-lowering effects, the results of the present study further support the role of Flu as a potential anti-fibrosis agent that may be a promising candidate for NASH therapy.

## Abbreviations

CDAA: Choline deficient L-amino acid defined
CM: Conditioned medium
FFAs: Free fatty acids
Fluvastatin: Flu
HSC: Hepatic stellate cell
NADPH: Nicotinamide adenine dinucleotide phosphate
NAFLD: Non-alcoholic fatty liver disease
NASH: Non-alcoholic steatohepatitis
NOX: NADPH oxidase
OS: Oxidative stress
PA: Palmitate
PRH: Primary rat hepatocyte
ROS: Reactive oxygen species
